# The Crumbs_C isoform of *Drosophila* shows tissue- and stage-specific expression and prevents light-dependent retinal degeneration

**DOI:** 10.1242/bio.020040

**Published:** 2017-02-08

**Authors:** Stephanie Spannl, Alexandra Kumichel, Sarita Hebbar, Katja Kapp, Marcos Gonzalez-Gaitan, Sylke Winkler, Rosana Blawid, Gregor Jessberger, Elisabeth Knust

**Affiliations:** 1 Max-Planck-Institute of Molecular Cell Biology and Genetics, Pfotenhauerstrasse 108, Dresden 01307, Germany; 2 Department of Biochemistry, Sciences II, University of Geneva, 30 Quai Ernest-Ansermet, Geneva 4 1211, Switzerland

**Keywords:** Epithelial polarity, EGF-like repeat, Alternative splicing, Mutually exclusive exon

## Abstract

*Drosophila* Crumbs (Crb) is a key regulator of epithelial polarity and fulfils a plethora of other functions, such as growth regulation, morphogenesis of photoreceptor cells and prevention of retinal degeneration. This raises the question how a single gene regulates such diverse functions, which in mammals are controlled by three different paralogs. Here, we show that in *Drosophila* different Crb protein isoforms are differentially expressed as a result of alternative splicing. All isoforms are transmembrane proteins that differ by just one EGF-like repeat in their extracellular portion. Unlike Crb_A, which is expressed in most embryonic epithelia from early stages onward, Crb_C is expressed later and only in a subset of embryonic epithelia. Flies specifically lacking Crb_C are homozygous viable and fertile. Strikingly, these flies undergo light-dependent photoreceptor degeneration despite the fact that the other isoforms are expressed and properly localised at the stalk membrane. This allele now provides an ideal possibility to further unravel the molecular mechanisms by which *Drosophila crb* protects photoreceptor cells from the detrimental consequences of light-induced cell stress.

## Abstract (precis)

**Summary:** Loss of Crb_C, one protein isoform encoded by *Drosophila crumbs*, results in light-dependent retinal degeneration, but does not affect any of the other *crumbs*-specific functions.

## INTRODUCTION

*Drosophila* Crumbs (Crb) is an evolutionarily conserved regulator of epithelial apico-basal polarity. Loss of *crb* function results in embryonic lethality, caused by the breakdown of many epithelia (Grawe et al., 1996; Tepass, 1996; Tepass et al., 1990). Besides a role in epithelial cell polarity, *Drosophila crb* controls tissue size in imaginal discs by acting upstream of the Hippo pathway (reviewed in Boggiano and Fehon, 2012; Genevet and Tapon, 2011; Sun and Irvine, 2016), regulates morphogenesis of photoreceptor cells, and prevents light-dependent retinal degeneration (reviewed in Bazellières et al., 2009; Bulgakova and Knust, 2009). Crb is a type I transmembrane protein, the extracellular portion of which is composed of an array of epidermal growth factor (EGF)-like repeats. Its small cytoplasmic tail of only 37 amino acids contains two conserved protein-protein interaction motifs, a C-terminal PDZ (PSD95/Discs large/ZO-1) domain-binding motif and a FERM (protein 4.1/ezrin/radixin/moesin) domain-binding motif. In epithelial cells, Crb is localised apically to the *zonula adherens* (ZA) where it organises a membrane-associated protein complex (reviewed in Bulgakova and Knust, 2009; Flores-Benitez and Knust, 2016; Le Bivic, 2013; Tepass, 2012).

All major components of the Crb protein complexes, their respective interactions, localisation and many of their functions are conserved in vertebrates. Mouse and human genomes contain three Crb genes, *Crb1/CRB1*, *Crb2/CRB2* and *Crb3/CRB3*, respectively, the first two of which are very similar to *Drosophila* Crb, while Crb3/CRB3 contains a very small and completely different extracellular region (Makarova et al., 2003; Roh et al., 2003). However, the cytoplasmic tails of all Crb proteins are highly conserved. Interestingly, functions that are covered by a single *crb* gene in *Drosophila* seem to be allocated to individual *Crb* genes in vertebrates. For example, mouse embryos mutant for *Crb2* die during gastrulation (Xiao et al., 2011), while human foetuses or zebrafish embryos carrying mutations in *CRB2/Crb2b*, respectively, develop renal defects and filtration impairment due to a failure to organise functional foot processes of the podocytes (Ebarasi et al., 2015; Slavotinek et al., 2015). Mice mutant for *Crb3* die shortly after birth, exhibiting cystic kidneys and defects in the lung and intestine (Charrier et al., 2015; Szymaniak et al., 2015; Whiteman et al., 2014). While mutations in human *CRB1* are associated with early-onset retinitis pigmentosa (RP12) and Leber congenital amaurosis (LCA) (den Hollander et al., 2001, 1999), it seems to be *Crb2* in the mouse that has taken on this function (Alves et al., 2014).

The specific functions of individual mammalian *Crb* paralogs raise the question of how a single gene in *Drosophila* can regulate a variety of functions during tissue development and homeostasis. It is obvious that some portions of the Crb protein are required for specific functions. The PDZ domain-binding motif of the cytoplasmic tail, for example, is of utmost importance for the development of most embryonic epithelia (Klebes and Knust, 2000; Klose et al., 2013; Wodarz et al., 1993), while the FERM domain-binding motif is required for dorsal closure in the embryo (Flores-Benitez and Knust, 2015; Klose et al., 2013) and participates in regulating the Hippo pathway in imaginal discs (Chen et al., 2010; Ling et al., 2010; Robinson et al., 2010). In contrast, the extracellular portion mediates growth regulation (Herranz et al., 2006; Richardson and Pichaud, 2010) and wing vein refinement (Nemetschke and Knust, 2016) via the Notch pathway, controls cell survival (Hafezi et al., 2012) and photoreceptor morphogenesis, and prevents light-dependent photoreceptor degeneration (Chartier et al., 2012; Izaddoost et al., 2002; Johnson et al., 2002; Pellikka et al., 2002; Richard et al., 2009). A second mechanism by which Crb achieves functional diversity is by recruiting different interaction partners in a stage- and/or tissue-specific manner (reviewed in Bulgakova and Knust, 2009; Flores-Benitez and Knust, 2016). Finally, alternative splicing can give rise to various protein isoforms, which may have different functions. For *Drosophila crb*, Flybase (http://flybase.org/, release 6.0) predicts four different Crb isoforms as a result of alternative splicing, Crb_A, which corresponds to the previously published isoform (Tepass et al., 1990; Wodarz et al., 1993), Crb_B, Crb_C and Crb_D. Recently, moderate overexpression of the Crb_C isoform in the embryo was linked to centrosome positioning defects similar to those induced by loss of the Ski-family helicase Obelus (Vichas et al., 2015).

Here we analyse the expression pattern of the predicted isoforms, and study the role of one of them, Crb_C, in more detail. Crb_C is expressed in a subset of embryonic epithelia and in adult photoreceptor cells. Flies carrying a mutation that specifically eliminates this isoform are homozygous viable and fertile. However, their photoreceptors undergo light-dependent degeneration, similar as photoreceptors of *crb* loss-of-function alleles, which lack all Crb isoforms (Chartier et al., 2012; Johnson et al., 2002). This raises the interesting possibility that it is Crb_C that protects photoreceptor cells from the damaging consequences of light-induced cell stress.

## RESULTS

### Crb isoforms differ by one EGF-like repeat

**Fig. 1.**
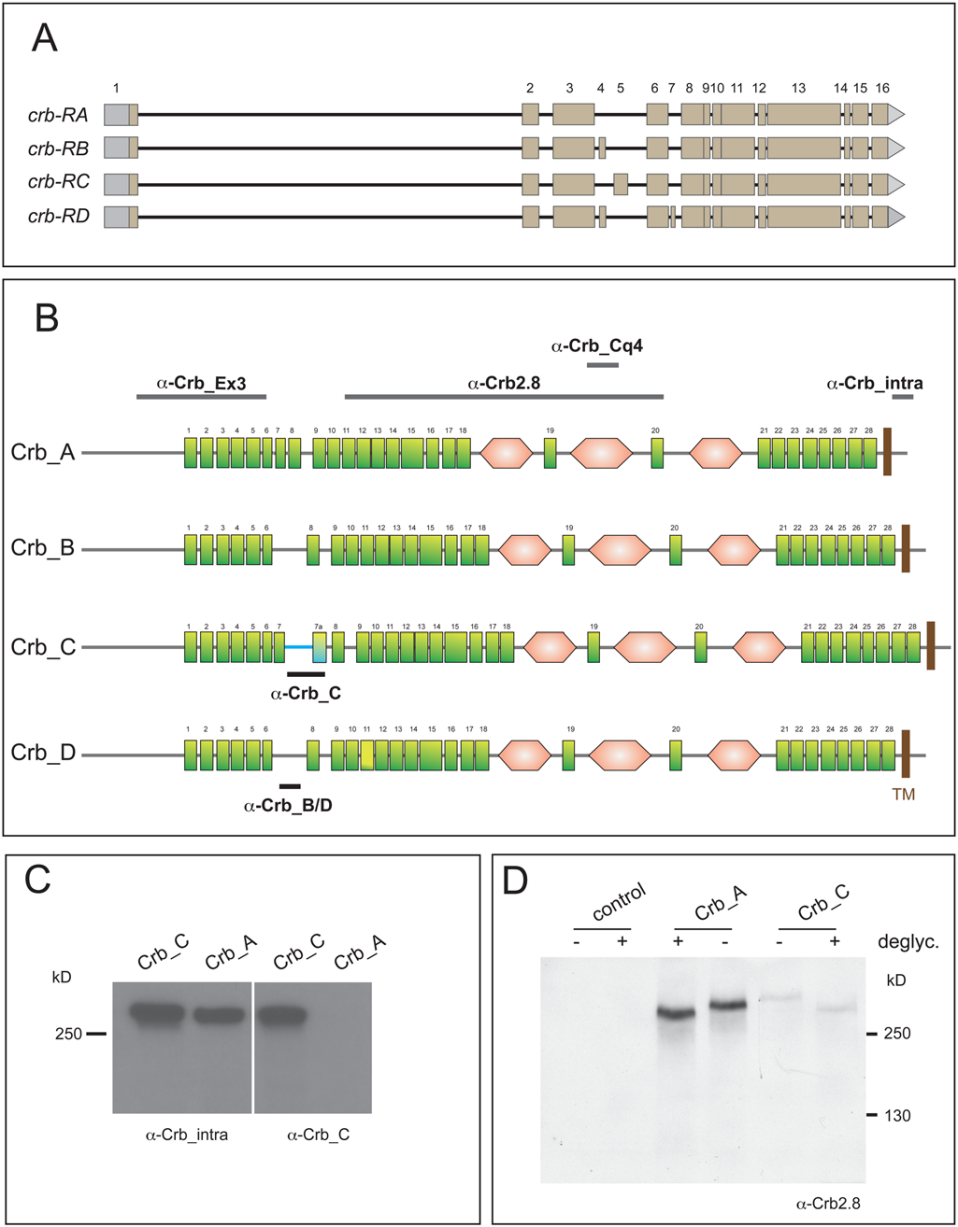
**The *crb* locus encodes four different protein isoforms.** (A) Four *crb* mRNA variants are the result of alternative splicing. Exons 1-3, 6 and 8-16 are shared. Exon 4 is specific for *crb-RB* and *crb-RD*, exon 5 is only found in *crb-RC* and exon 7 only in *crb-RD*. Grey boxes indicate 5′ and 3′ UTRs. (Adapted from Flybase.) (B) The four predicted Crb isoforms. Green rectangle: EGF-like repeat (numbering based on Crb_A), pink hexagon: repeat with similarity to the globular domain of laminin A, TM: transmembrane domain. The four isoforms have the same N-terminus, including EGF-like repeat #1-6, and share the C-terminus from EGF-like repeat #8 onwards, except for EGF-like repeat #11, which carries a 12 amino acid insertion in Crb_D. Isoforms B and D lack EGF-like repeat #7, isoform C carries an additional EGF-like repeat (#7a). The horizontal bars indicate the regions used as epitopes for immunization. Antibodies indicated on top detect all isoforms. α-Crb_C is specific for isoform C, α-Crb_B/D is specific for isoforms B and D. The blue line in isoform C indicates the isoform-specific insertion (see also Fig. S1). (The current version of SMART used does not suggest an EGF-like repeat between #8 and #9, nor an additional laminin A-like repeat N-terminal to EGF-like repeat #1 as shown in some previous publications.) (C) Western blot to demonstrate the specificity of α-Crb_C antibody. Crb_A and Crb_C were overexpressed in *Drosophila* S2R^+^ cells and cell extracts were probed with either α-Crb_intra, which detects both isoforms, or α-Crb_C. Upon strong overexpression, α-Crb_C detects two bands, which presumably represents the non-glycosylated precursor and the mature glycoprotein, as upon deglycosylation only a single band is detected (shown in D). (D) Crb_A and Crb_C were overexpressed in *Drosophila* S2R^+^ cells, cell extracts were deglycosylated and probed with α-Crb2.8. Crb_C runs slightly slower than Crb_A, independent of whether it is glycosylated or not.

The *crumbs* (*crb*) locus of *Drosophila melanogaster*, named after its embryonic cuticle phenotype (Jürgens et al., 1984), encodes a single-pass type I transmembrane protein (Kilic et al., 2010; Tepass et al., 1990; Wodarz et al., 1993). Flybase (http://flybase.org/) predicts four different isoforms, Crb_A, Crb_B, Crb_C and Crb_D, which are the result of alternative splicing of the *crb* pre-mRNA. The predicted *crb-RB* differs from the previously published *crb-RA* mRNA by the presence of an additional exon of 129 nucleotides between the common exons 3 and 6 (exon 4), while the predicted *crb-RC* carries an additional exon of 321 nucleotides (exon 5) (Fig. 1A). The predicted *crb-RD* mRNA contains exon 4 as the predicted *crb*-RB mRNA and a further exon (exon 7, 42 nucleotides) localised between the common exon 6 and 8 (Fig. 1A).

All predicted Crb isoforms are transmembrane proteins, which share an unusually long signal peptide (Kilic et al., 2010), an array of EGF-like repeats interspersed by three repeats with similarity to the globular domain of laminin A, a transmembrane domain, and a short cytoplasmic tail of 37 amino acids (Fig. 1B). The four predicted isoforms differ in the number of EGF-like repeats, which is 27 (Crb_B, Crb_D), 28 (Crb_A) and 29 (Crb_C) [according to ProSite (http://prosite.expasy.org/) and SMART (http://smart.embl-heidelberg.de/)] (Fig. 1B). Exon 4 in *crb-RB* and *crb-RD* introduces 43 amino acids (grey box in Fig. S1), thereby eliminating EGF-like repeat #7. The additional exon 7 in *crb-RD* adds a stretch of 14 amino acids into EGF-like repeat #11 of Crb_D (Fig. S1, yellow box). Exon 5 in *crb-RC* inserts an array of 107 amino acids into EGF-like repeat #7 (Fig. S1, blue letters). The inserted amino acid sequence completes EGF-like repeat #7 and adds one more EGF-like repeat (#7a), which terminates with the same four amino acids as EGF-like repeat #7 of Crb_A. The sequence between EGF-like repeat #7 and #7a in Crb_C is unusually rich in threonine and proline residues. NetOGlyc 3.1 (http://www.cbs.dtu.dk/services/NetOGlyc-3.1/) predicts the threonine residues to represent sites of mucin-type O-glycosylation as posttranslational modification (Julenius et al., 2005; Tran and Ten Hagen, 2013). In fact, this stretch carries 19 predicted mucin-type O-glycosylation sites, while the rest of the Crb protein, which is shared by all isoforms, contains only a single one located in the third laminin A-like repeat (M. Eichel, Knust Lab, MPI-CBG Dresden, Germany and K. K., unpublished).

### Differential expression of three predicted Crb isoforms in *Drosophila* embryos

*crb* RNA and Crb protein are supplied maternally (Laprise et al., 2006), and zygotic *crb* transcription starts at stage 6 and continues throughout embryogenesis (Tepass et al., 1990). As revealed by RT-PCR, only *crb-RA* and *crb-RC*, but not *crb-RB* and *crb-RD* mRNAs were detected in the embryo (data not shown). To further characterise the expression pattern of the corresponding proteins, we raised antibodies against exon 4 (called α-Crb_B/D) and exon 5 (called α-Crb_C), which specifically recognise the Crb_B/D isoforms and the Crb_C isoform, respectively (Fig. 1B). In addition, we used four antibodies which recognise all isoforms: the previously published polyclonal antibody α-Crb2.8 (Tepass et al., 1990), the monoclonal antibody α-Crb_Cq4 (Tepass and Knust, 1993), the polyclonal antibody α-Crb_intra2662 raised against the cytoplasmic tail (Kumichel et al., 2015), and a newly raised antibody against the common exon 3 (α-Crb_Ex3) (Fig. 1B). The specificity of the newly generated antibodies was confirmed by the lack of staining in *crb^11A22^* homozygous mutant embryos (data not shown). The specificity of the α-Crb_C antibody was further tested by western blot analysis of recombinant Crb_A and Crb_C proteins, overexpressed in S2R^+^ cells. α-Crb_C antibody only recognises recombinant Crb_C protein, but not Crb_A, while α-Crb_intra2662 detects both proteins (Fig. 1C). Both overexpressed isoforms are glycosylated, and Crb_C migrates slightly slower than Crb_A (Fig. 1D), which is consistent with the insertion encoded by exon 5.

**Fig. 2.**
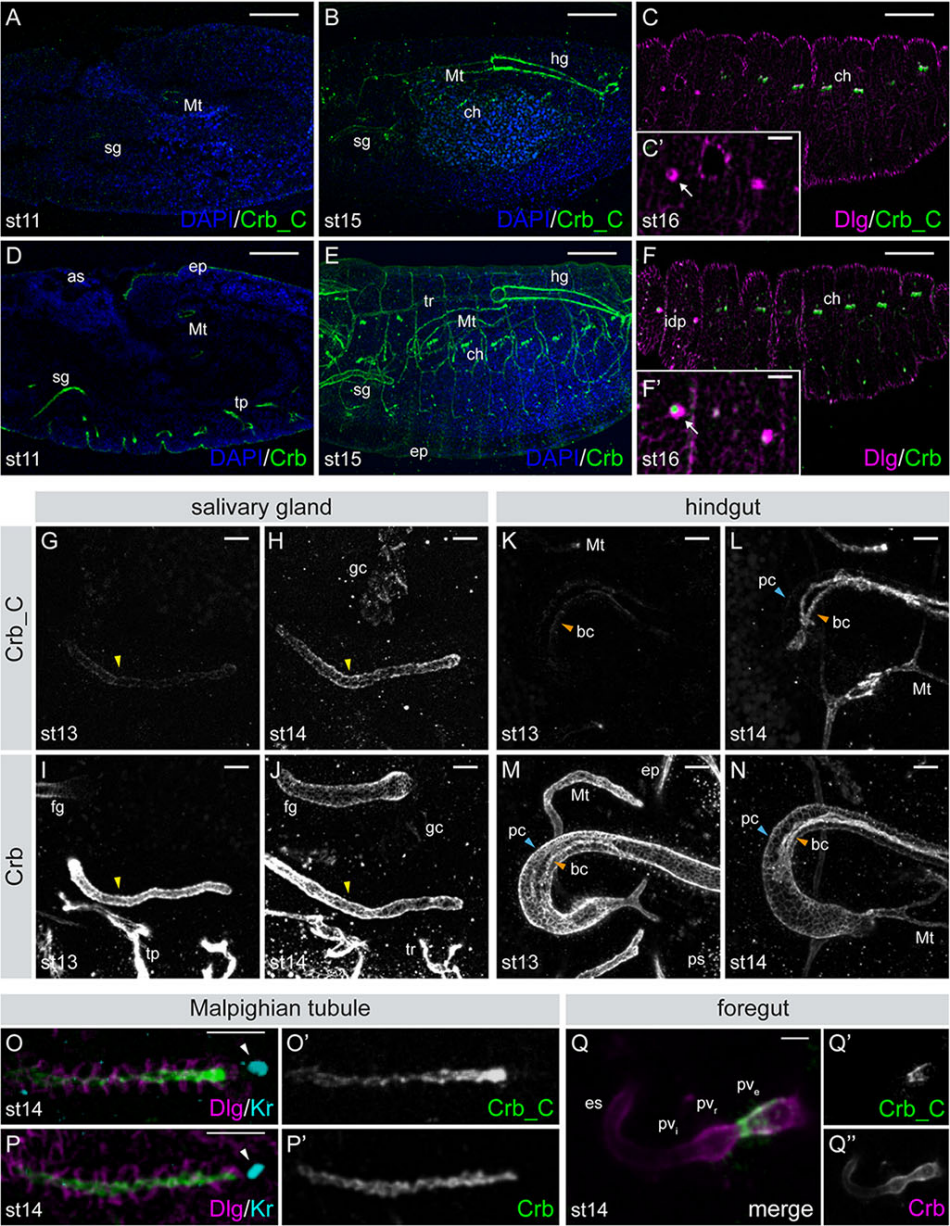
**Expression of Crb isoforms during embryogenesis.** (A-F) Lateral view of wild-type embryos at stage 11 (A,D), stage 15 (B,E), and stage 16 (C,F) stained with α-Crb_C or α-Crb (green) and α-Discs large (Dlg; magenta); nuclei (DAPI, blue). Scale bars: 50 µm. (C′,F′) Higher magnification of the precursors of the imaginal disc (idp; arrow). Scale bars:10 µm. (G-N) Dorsal views of stage 13 and 14 embryos showing the salivary gland (sg; G-J, yellow arrowheads) and hindgut (hg; K-N). Crb_C expression increases during sg development. In the hg, expression of Crb_C is first detected in the boundary cells at stage 13 (orange arrowheads in K-N). Crb_C expression level gradually increases during embryogenesis (L). The principal cells (pc; blue arrowheads) express very low levels of Crb_C (L). (O,P) Dorsal view of the Malpighian tubules (Mt) at stage 14 stained with α-Crb_C (O,O′) or α-Crb (P,P′). Dlg (magenta) marks the lateral membranes, Krüppel (Kr, cyan) the nucleus of the distal tip cell (white arrowheads). Crb_C is predominantly expressed at the distal tip. (Q) Ventral view of the foregut stained with α-Crb_C (green, Q′) and α-Crb (magenta, Q″). α-Crb_C only stains the external portion of the proventriculus (pv_e_), while α-Crb stains all parts. as, amnioserosa; bc, boundary cells; ch, chordotonal organs; ep, epidermis; es, esophagus; fg, foregut; gc, garland cells; hg, hindgut; idp, imaginal disc precursors; Mt, Malpighian tubule; pc, principal cells; ps, posterior spiracle; pv_e_, external portion of the proventriculus; pv_i_, internal portion of the proventriculus; pv_r_, recurrent portion of the proventriculus; sg, salivary gland; tp, tracheal pits; tr, tracheal tree. Anterior to the left. Scale bars: 10 µm.

The Crb antibodies as described above were used to analyse the expression pattern and localisation of the different Crb isoforms in *Drosophila* embryos. In agreement with RT-PCR data, the antibody against Crb_B/D did not recognise any epitope in wild-type embryos (Fig. S2C,C′). Therefore, the common antibodies (α-Crb_Cq4, α-Crb_intra2662 and α-Crb_Ex3) detect both Crb_A and Crb_C in the embryo, while α-Crb_C antibody is isoform-specific. While α-Crb2.8 detects Crb protein from stage 6 onwards (Tepass et al., 1990), the first faint expression of Crb_C was detected only in late stage 11 embryos in the salivary glands and the Malpighian tubules (Fig. 2A). Expression of Crb_C gradually increased in these tissues until completion of germ band retraction, and was additionally detected in the hindgut, the chordotonal organs (Fig. 2B,C) and part of the foregut (Fig. 2Q). In contrast, α-Crb2.8 recognised Crb protein at stage 11 in the amnioserosa, the epidermis, the Malpighian tubules, the salivary glands, the tracheal pits (Fig. 2D), and the fore- and hindgut (Fig. 2E,M,N, and data not shown). Staining with α-Crb2.8 revealed high protein expression in these tissues during germ band shortening and later on (Fig. 2E), and in the chordotonal organs, the tracheal tree (Fig. 2E,F) and the anterior and posterior spiracles (Fig. 2M and data not shown).

Additional similarities and differences between Crb_A and Crb_C expression were observed. During head involution and dorsal closure (stages 14/15), the expression pattern of Crb_C was maintained, with the salivary glands, the hindgut and the Malpighian tubules showing elevated expression levels (Fig. 2G-N). In addition, both Crb_A and Crb_C were detected in the embryonic garland cells (Fig. 2H,J), which function as nephrocytes. At the end of embryogenesis, the proventriculus, the hindgut, chordotonal organs, garland cells, salivary glands and Malpighian tubules expressed Crb_C. α-Crb2.8 detected Crb proteins in these tissues as well, and additionally recognised Crb protein in the epidermis, the tracheae (Fig. 2E), the pharynx, the esophagus (not shown) and the anlagen of the imaginal discs (Fig. 2F′).

Previously published data show Crb protein expression in the embryonic hindgut as soon as the primordium of the hindgut is formed (Tepass et al., 1990). Crb_C expression, however, was first detected in the hindgut at stage 13, where it first became apparent in the boundary cells, two cell rows separating the dorsal and ventral compartment of the hindgut (Fuss and Hoch, 1998; Iwaki and Lengyel, 2002; Kumichel and Knust, 2014; Tepass et al., 1990). Crb_C expression level in the boundary cells increased as embryogenesis proceeded, whereas the majority of hindgut cells, the principal cells, were hardly labelled with α-Crb_C (Fig. 2K,L). In contrast, α-Crb2.8 clearly detected Crb protein in both the boundary and principal cells at stage 13 and later (Fig. 2M,N). In the Malpighian tubules, Crb_C was expressed in a graded fashion, with the highest expression in the distal tip cell (Fig. 2O,O′). In contrast, α-Crb2.8 revealed uniform apical expression (Fig. 2P,P′). Another difference was obvious in the embryonic foregut. While α-Crb_Ex3 and α-Crb2.8 stained all three parts of the foregut, i.e. the pharynx, esophagus and proventriculus from stage 14 onwards, Crb_C was only expressed in the posterior part of the proventriculus, the so-called external portion or outer layer of the proventriculus (Fig. 2Q).

Taken together, Crb_C exhibits a different spatio-temporal expression pattern in the embryo in comparison to Crb_A. Crb_C expression starts later during *Drosophila* embryogenesis and gradually increases as development proceeds, and its expression is restricted to a subset of tissues, most of them tubular organs (summarised in Table S1). Similar to Crb_A, Crb_C is localised apically in all epithelia where it is expressed.

### Localisation of Crb_C in *crb* and *sdt* mutant *Drosophila* embryos

According to earlier studies, Crb and the scaffolding protein Sdt interact through the C-terminal PDZ domain-binding motif of Crb and the PDZ domain of Sdt (Bachmann et al., 2001; Hong et al., 2001). In most tissues, this interaction is required to mutually stabilise Crb and Sdt at the apical membrane. To find out whether this interaction is also required for apical localisation of Crb_C, we analysed Crb_C localisation in embryos mutant for either *crb^8F105^* or *sdt^K85^*. *crb^8F105^* encodes a truncated Crb protein lacking the C-terminal 23 amino acids, including the PDZ domain and hence the Sdt binding site, and behaves like a complete loss-of-function allele in the embryo (Wodarz et al., 1993). The amorphic allele *sdt^K85^* carries a premature stop codon in the N-terminal L27 domain and affects all known Sdt isoforms (Berger et al., 2007).

**Fig. 3.**
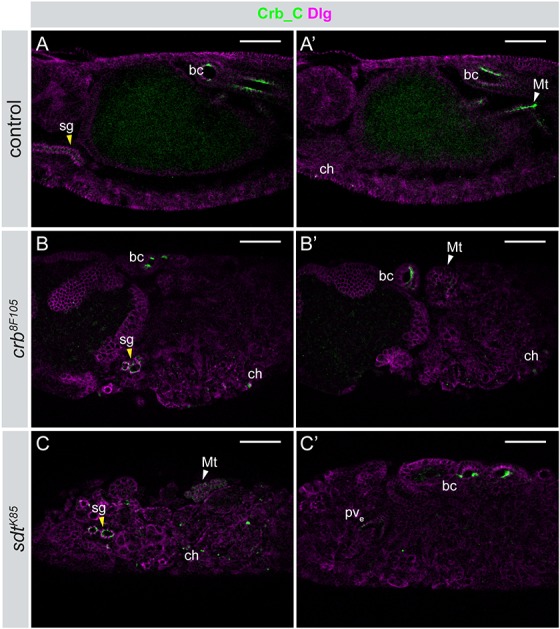
**Apical localisation of Crb_C is independent of the Crb/Sdt interaction in *Drosophila* embryos.** Different optical sections of control (*crb^GX24^*/+) (A/A′), homozygous *crb^8F105^* (B/B′) and *sdt^K85^* (C/C′) embryos stained with α-Crb_C (green) and α-Dlg (magenta). Crb_C localizes normally in the boundary cells (bc), the chordotonal organs (ch) and the salivary glands (sg; yellow arrowhead in A,B, and C) in both mutants, whereas it is delocalised in the Malpighian tubules (Mt; white arrowhead in A′,B′ and C). pv_e_, external portion of the proventriculus. Anterior is to the left. Scale bars: 50 μm.

In *crb^8F105^* and *sdt^K85^* mutant embryos, some epithelia, such as the epidermis, exhibit a complete breakdown of tissue integrity, while others, such as the hindgut, the rudimentary salivary glands and the proventriculus maintain aspects of cell polarity (Grawe et al., 1996; Kumichel and Knust, 2014; Tepass, 1996; Tepass and Knust, 1990, 1993). In epithelia that maintain polarity, Crb_C was still restricted to the apical side, as seen in the rudimentary salivary glands, the proventriculus and the boundary cells of the hindgut (Fig. 3B,B′,C,C′). No signal could be detected in the principal hindgut cells of *crb^8F105^* and *sdt^K85^* mutant embryos when using α-Crb_C, similar as has been shown for α-Crb_Ex3 or α-Crb_Cq4 (Kumichel and Knust, 2014). Crb_C was still normally expressed in chordotonal organs, which are dislocated due to the breakdown of the epidermal tissue structure (Fig. 3B,B′,C). Malpighian tubules fail to elongate and appear as disorganised cell clusters in both mutants (Campbell et al., 2009). Crb_C was expressed in the Malpighian tubules, but was diffusely distributed (Fig. 3B′,C and data not shown).

Taken together, these data indicate that at late stages of embryogenesis, localisation and stabilisation of Crb_C at the apical membrane is independent of a functional Crb-Sdt interaction in those tissues that maintain aspects of apico-basal polarity. Due to the lack of a Crb_A-specific antibody, we currently do not know whether this behaviour is specific for Crb_C or also applies for Crb_A in these tissues. In *crb* and *sdt* mutant embryos, the tracheal system breaks down, but individual epithelial cysts maintain apico-basal polarity (Tepass and Knust, 1990, 1993). Crb_A, the only isoform expressed in this tissue, was not apically localised in these cysts (data not shown), showing that its localisation requires a functional Crb-Sdt interaction, at least in this tissue.

### Expression of Crb isoforms in larval tissues

**Fig. 4.**
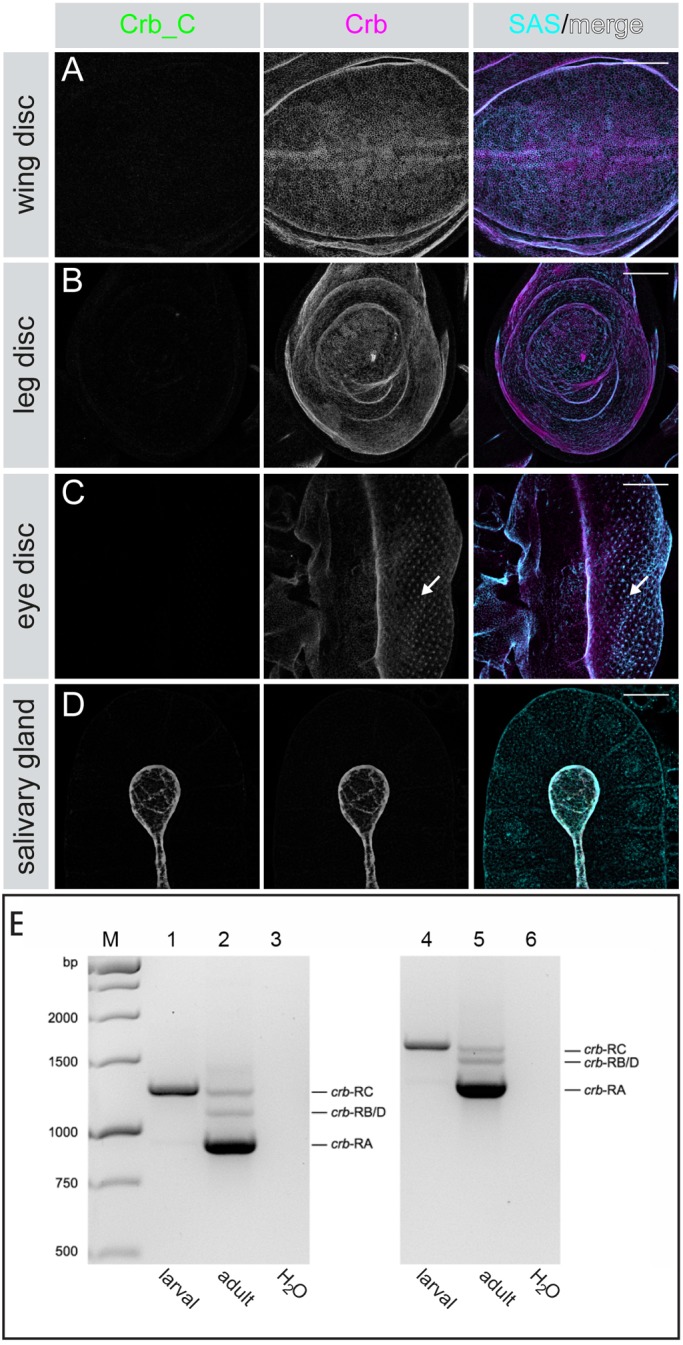
**Postembryonic expression of Crb isoforms.** (A-D) Expression of Crb isoforms in tissues of wild-type third instar larvae stained with α-Crb_C (green), α-Crb_Ex3 (magenta), and α-Stranded at Second (SAS, cyan) as apical marker. Crb localises to the apical membrane. In the eye disc, Crb is predominantly expressed behind the morphogenetic furrow, and is strongly enriched at the apical membranes of differentiating photoreceptor cells (arrow in C). Crb_C expression is not detected in the examined imaginal discs (A-C), but is observed in the larval salivary gland (D). Scale bars: 50 µm. (E) Gel electrophoresis of RT-PCR experiments to identify transcribed *crb* mRNAs. Larval, RNA from larval large intestine; adult, RNA from adult flies; H_2_O, negative control (no DNA). For primers used, see Table S2. M: 1 kb DNA Ladder. 1, 2, 3: primer pair 1, expected sizes *crb-RA* 919 bp, *crb-RB/D* 1048 bp, *crb-RC* 1240 bp. 4, 5, 6: primer pair 2, expected sizes *crb-RA* 1285 bp, *crb-RB/D* 1414 bp, *crb-RC* 1606 bp.

Crb is expressed at postembryonic stages in several tissues, such as the larval salivary gland and imaginal discs (Tepass and Knust, 1990), which represent the anlagen of most of the external structures of the fly, e.g. the wing, the legs or the eye. Cells of the imaginal discs are set-aside during late stages of embryogenesis and start to proliferate only in larval stages. As shown above, α-Crb2.8, but not α-Crb_C, detected Crb protein in the anlagen of the imaginal discs in stage 16/17 embryos (Fig. 2F′). This expression pattern seemed to be maintained throughout larval development, since α-Crb_Ex3 gave a strong signal in the eye-, leg- and wing imaginal discs of third instar larvae. In contrast, Crb_C was not detected in these discs (Fig. 4A-C), while Crb_B/D was absent in leg and wing discs, but could be detected in developing photoreceptor cells in eye discs (data not shown). Similar as in the embryo, the salivary glands of third instar larvae expressed Crb_C (Fig. 4D). In the larval hindgut, α-Crb2.8 antibody staining showed uniform staining on the entire apical membrane (data not shown). Unfortunately, staining with the α-Crb_C antibody did not work in the larval hindgut. Therefore, RT-PCR was performed from RNA isolated from the hindgut of third instar larvae to reveal which isoform is expressed. This experiment demonstrated that the *crb-RC* mRNA was the predominant one expressed in the larval hindgut (Fig. 4E).

### Flies lacking the Crb_C isoform undergo light-dependent retinal degeneration

Crb_C expression in embryonic and larval tissues is clearly distinct from that of Crb_A, leading to the question of whether the specific loss of this isoform also results in distinct phenotypes. To unravel the function of the Crb_C isoform, we isolated mutations by TILLING (targeting induced local lesions in genomes). In total, we screened 2400 genomes for variants in the *crb-RC*-specific amplicon. We identified two genomes with sequence differences resulting in nonsense mutations. Lines *crb^p17F5^* and *crb^p13A9^* carry CGA to TGA mutations, thus changing R575 and Q590 of the Crb_C isoform to a stop codon, respectively (Fig. S1). As a consequence, mutant flies should express truncated Crb_C variants, but leave Crb_A and Crb_B/D unaffected. The mutant lines were recovered from the living fly library and crossed for four generations to w* flies to reduce the number of associated sequence variants. Strikingly, *crb^p17F5^* and *crb^p13A9^* mutant flies were viable and fertile as homo- and hemizygotes and in trans over the amorphic allele *crb^11A22^*. They did not show any obvious developmental or morphological abnormalities. No differences in larval hatching rate and in adult life span were observed for *crb^p13A9^*. In contrast, life span of homozygous mutant adult *crb^p17F5^* flies was reduced, which is probably due to the genetic background, since *crb^p17F5^/crb^p13A9^* did not show any significant deviation in lifespan compared to that of wild-type flies (data not shown). In accordance with the molecular data, α-Crb_C did not detect any protein in *crb^p13A9^* homozygous mutant embryos (Fig. S2A-B′). Expression of Crb_A was not affected in *crb^p13A9^* mutant embryos (Fig. S2A-D′) and Crb_B/D was still absent (Fig. S2C-D′).

**Fig. 5.**
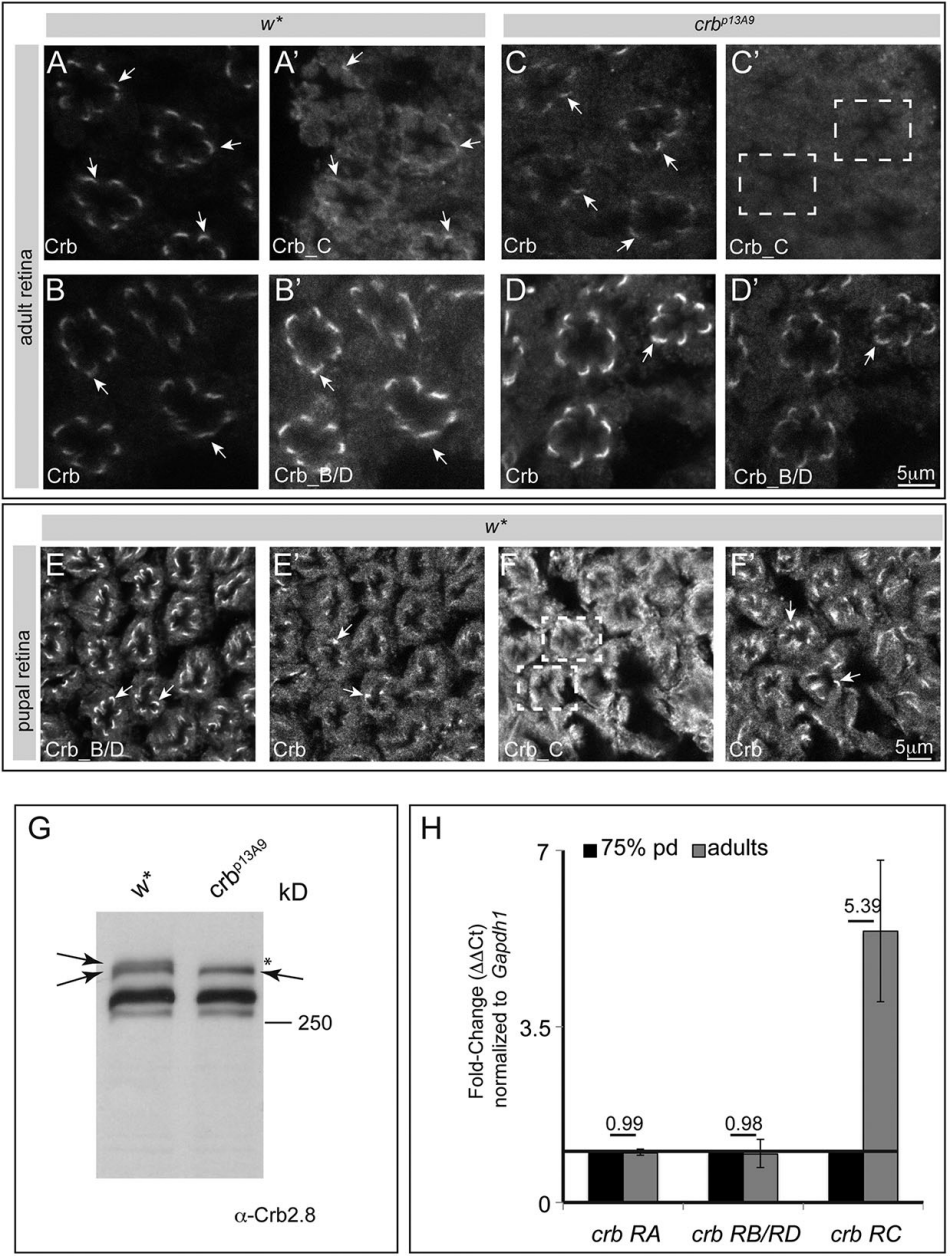
**Expression of Crb_C in pupal and adult wild-type and mutant flies.** (A-F′) Confocal images of representative optical sections from retinal whole mounts of adult (1 day old female flies; A-D′) and pupae (72 h APF; E-F′) from w* and *crb ^p13A^*^9^ animals, stained for Crb. (A-D) α-Crb_Cq4 detects all isoforms, α-Crb_B/D is specific for Crb_B/D and α-Crb_C is specific for Crb_C. Arrows indicate stalk membrane of PRCs. Dashed white boxes in C′ and F indicate ommatidial clusters with no obvious α-Crb_C immunoreactivity at stalk membranes. Scale bar: 5 μm. (G) Western blot of protein extracts isolated from control (w*) and *crb^p13A9^* homozygous mutant adult heads (2 days old female flies), probed with α-Crb2.8, detecting all isoforms. The upper arrow points to a slower migrating protein present in head extracts of wild-type flies, which is missing in the mutant (*). The lower arrow points to a protein that is detected in w* and *crb^p13A9^*, which co-migrates with overexpressed Crb_A (data not shown). The identity of the other bands cannot unambiguously be determined. (H) Graph depicts fold-change (on *y*-axis, quantified as Δ Δ*Ct*) after normalisation with housekeeping gene *Gapdh1*, for different *crb* transcripts (on *x*-axis) from heads between 72 h APF (pupal stages) and newly eclosed adult. Whilst there is negligible change in *crb-RA* (fold-change=0.99) and *crb-RB/D* transcripts (fold-change=0.98), *crb-RC* transcript levels increase by 5.39-fold between the last day of pupal development and at eclosion (72 h APF and adulthood). Error bars depict s.e.m.

Beside various roles in embryos and larvae, *crb* has important functions during photoreceptor (PRC) development and homeostasis. Therefore, we analysed the expression of the different Crb isoforms in PRCs and looked for any mutant phenotype in eyes of *crb^p17F5^* and *crb^p13A9^* flies. As previously shown using an antibody that detects all Crb variants (α-Crb_Cq4), Crb localisation in adult PRCs is restricted to the stalk membrane, the portion of the apical membrane between the ZA and the rhabdomere (Fig. 5A,B, arrows). Similarly, both α-Crb_C and α-Crb_B/D labelled the stalk membrane (Fig. 5A′-B′, arrows), suggesting that all Crb isoforms are expressed in adult PRCs. Deep sequencing experiments revealed that *crb-RC* is the predominant *crb* mRNA expressed in head tissue and specifically in eyes (Fig. S3). In PRCs of *crb^p13A9^* mutant flies no signal was detected with α-Crb_C, while both α-Crb_B/D and α-Crb_Cq4 detected Crb proteins at the stalk membrane (Fig. 5C-D′). Absence of Crb_C in heads of adult *crb^p13A9^* mutant flies was confirmed by western blot (Fig. 5G). Since Crb_C was expressed in adult eyes, but not in larval eye imaginal discs, we analysed its expression in pupal eyes. Both Crb_C as well as Crb_B/D could be detected in PRCs at 72 h after puparium formation (h APF), consistent with previous observations using α-Crb2.8 (Fig. 5E-F′) (Pellikka et al., 2002; Richard et al., 2006). At this time, PRCs have achieved their adult morphology, i.e. they have formed distinct polarised membranes. The disparity in expression of these Crb isoforms between 72 h APF and adulthood is also reflected at the level of their respective transcripts. Quantitative RT-PCR (qRT-PCR) analyses revealed a more than fivefold increase in *crb-RC* levels in the head of flies shortly after eclosion in comparison to heads of 72 h APF pupae (Fig. 5H). In contrast, *crb-RA* and *crb-RB/RD* levels remained relatively unchanged during this period.

**Fig. 6.**
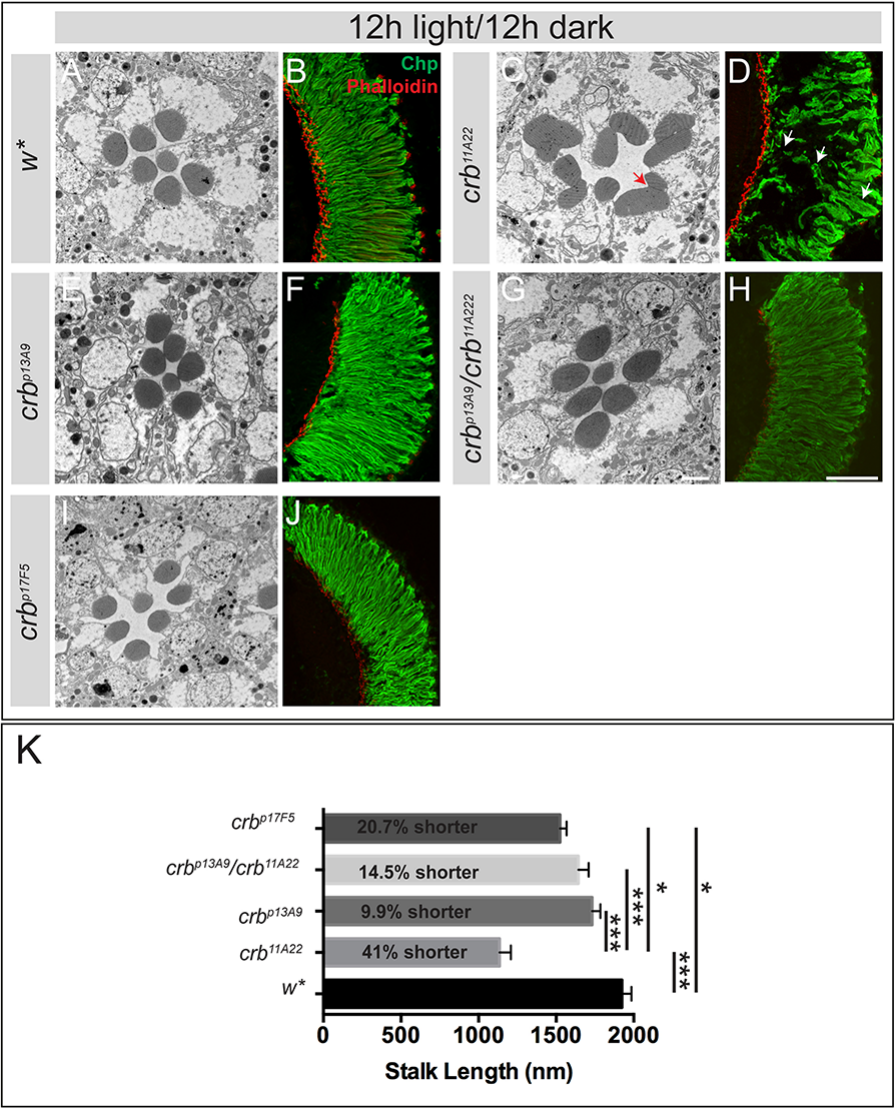
***crb_C* mutant photoreceptor cells exhibit normal morphology.** (A-J) Representative TEM images of retinal cross sections (A,C,E,G,I) and confocal images of longitudinal retinal sections (B,D,F,H,J) stained for Chaoptin (green) and Phalloidin (red) of adult flies, prepared 2 days after eclosion (light/dark cycle). *crb^11A22^* show mosaic eyes, all other eyes were from flies of the indicated allele/allelic combination. Fused rhabdomeres (red arrow) and incompletely elongated rhabdomeres (white arrows) are only seen in *crb^11A22^* mosaic retinas. Scale bars: 1.7 µm (A,C,E,G,I), 50 µm (B,D,F,H,J). (K) Mean stalk length (nm)±s.e.m. of PRCs of different genotypes. Statistically significant changes between genotypes (highlighted by a black line) are indicated with ***P<0.05E-10 and *P<0.05E-4 following ANOVA and post hoc Bonferroni test. The average reduction in stalk length is 20.7% for *crb^p17F5^*, 14.5% for *crb^p13A9^/crb^11A22^*, 9.9% for *crb^p13A9^*, and 41% for *crb^11A22^* with respect to the genetic control (w*).

**Fig. 7.**
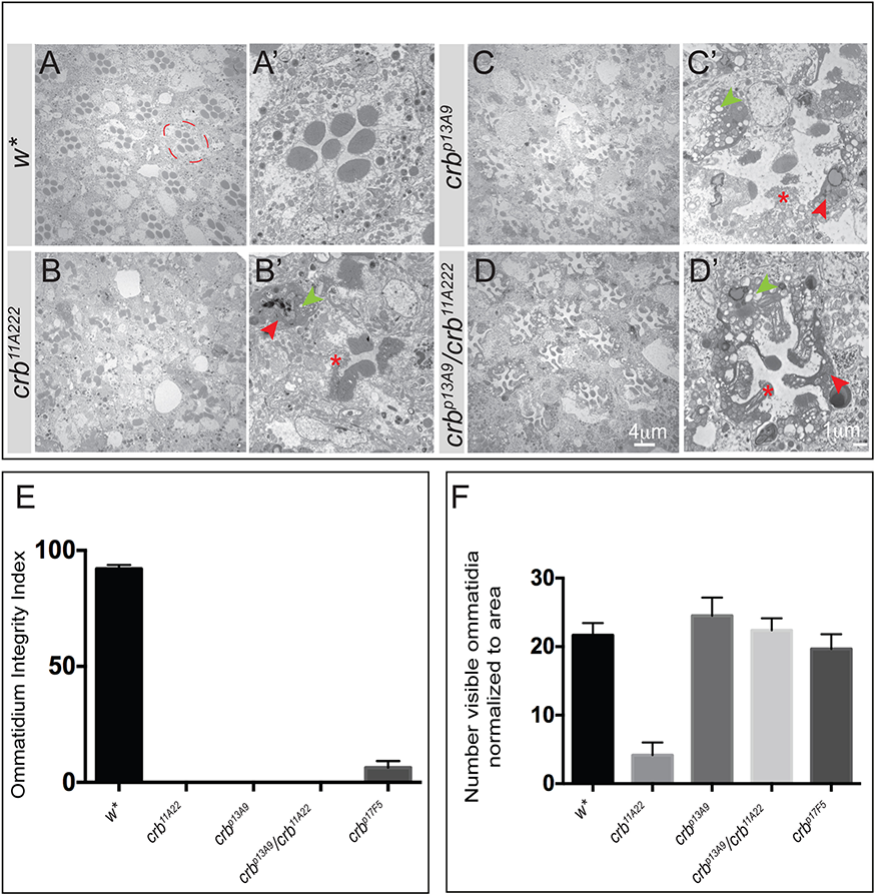
***crb_C* mutant photoreceptor cells undergo light-dependent degeneration.** (A-D′) Representative TEM images of retinal cross sections of adult flies of the indicated genotypes after 7 days continuous light exposure. A-D are overview images, A′-D′ are higher magnifications of one ommatidium. The stereotypic trapezoid arrangement of the seven rhabdomeres (w*) is lost in all *crb* alleles. Signs of degeneration include loss of rhabdomeric integrity (red asterisk), accumulation of electron dense debris (red arrowheads) and extensive vacuolization (green arrowheads). The dashed red circle in A outlines one ommatidium. Scale bars: 4 µm (A,B,C,D), 1 µm (A′,B′,C′,D′). (E) Bar graph depicting the mean Ommatidium Integrity Index (OII)±s.e.m. Ommatidial integrity index is the percent ratio of ommatidia (with no obvious signs of intracellular debris and vacuolization) to the total number of ommatidia normalized to area for the genotypes indicated on the *x*-axis. The significantly reduced OII in all *crb* alleles highlights retinal degeneration in these genotypes. (F) Bar graph depicting the mean number±s.e.m. of remnant ommatidia, normalized to area, in each of the genotypes indicated on the *x* -axis. Although there is degeneration in all *crb* mutant alleles, the phenotype is most severe in the loss-of-function allele *crb^11A22^* (large clone mosaics), evident from the reduced number of identifiable ommatidia per unit area (compare with sections shown in the TEM panel above). Sample size consists of three biological replicates, from which at least 100 ommatidia were evaluated (with the exception of *crb^11A22^*, in which most of the ommatidia completely degenerate and only 25 ommatidia could be identified for quantification).

Complete loss of *crb* function affects PRC morphogenesis. Rhabdomeres are thicker, frequently contact adjacent rhabdomeres and do not reach the base of the retina in most cases, and the stalk is reduced in length. When exposed to constant light, *crb* mutant PRCs undergo retinal degeneration (Chartier et al., 2012; Izaddoost et al., 2002; Johnson et al., 2002; Pellikka et al., 2002). Therefore, we examined the morphology of PRCs lacking Crb_C shortly after eclosion and after 7 days of continuous light exposure, and compared it to that of control and *crb^11A22^* mutant eyes (Figs 6 and 7). Upon eclosion, neither *crb^p13A9^* (Fig. 6E,F), nor *crb^p13A9^*/*crb^11A22^* (Fig. 6G,H), nor *crb^p17F5^* (Fig. 6I,J) mutant PRCs displayed any morphogenetic phenotypes. Their overall PRC morphology is comparable to the genetic control examined, w* (Fig. 6A,B). *crb^p17F5^* showed a mild (20%) decrease in stalk length, which was, however, not as severe as in *crb^11A22^* (decrease of 41%; Fig. 6K). This indicates that Crb_C does not play a major role in PRC morphogenesis. However, upon exposure to constant light, PRCs of the two newly generated alleles *crb^p13A9^* (Fig. 7C,C′) and *crb^p17F5^* (data not shown) and of the trans-heteroallelic combination of *crb^p13A9^* and *crb^11A22^* (Fig. 7D,D′) showed clear signs of degeneration, similar as PRCs of the loss-of-function allele *crb^11A22^* (Fig. 7B,B′) (Chartier et al., 2012; Johnson et al., 2002). Degeneration is characterised by the loss of rhabdomeric (apical) membrane integrity, accumulation of electron-dense debris, and extensive vacuolisation in the PRCs (Fig. 7). To quantify the results, we determined ommatidial integrity, measured as percent of remnant ommatidia with no obvious signs of degeneration, to total ommatidia, and normalised it to area (Fig. 7E). Whereas in w* retinas almost all ommatidia were intact (92%±3.5), this number was reduced to 6.3%±3.5 in *crb^p17F5^* and to 0 in homozygous *crb^p13A9^* and *crb^11A22^* and in transheterozygous *crb^p13A9^/crb^11A22^* PRCs. Interestingly, the absolute number of discernibly remnant ommatidia was lower in the case of the loss-of-function allele *crb^11A22^* (Fig. 7B, quantified in Fig. 7F). From this we conclude that *crb^p17F5^* and *crb^p13A9^* mutant PRCs still have some residual *crb* function.

## DISCUSSION

Here, we present data to show that three of the four predicted proteins encoded by *Drosophila crb* are differentially expressed during development. We focussed on Crb_A and Crb_C, the two isoforms expressed during embryogenesis. Crb_C comes up later than Crb_A and is expressed only in a subset of epithelia, namely the salivary glands, the Malpighian tubules, the boundary cells of the hindgut and part of the foregut (summarized in Table S1). In addition, Crb_C could be detected in the chordotonal organs and the garland cells. Crb_C is not expressed in the tracheae, in the epidermis and the amnioserosa. In embryos lacking Crb_C, expression of Crb_A is not changed and Crb_B/D is still not expressed.

What do tissues expressing Crb_C have in common? Most of them are tubular organs, including the ommatidium, which forms the interrhabdomeral space (IRS). Furthermore, most Crb_C-expressing tissues develop various membrane protrusions, which are required for absorption (boundary cells), endocytosis (garland cells), sensing (chordotonal organs, PRCs) or secretion (outer layer of proventriculus, salivary glands, Malpighian tubules) (see Table S1), but not needed before the larvae hatch. Their function may be compromised in larvae lacking Crb_C, but still work sufficiently well to allow survival under standard laboratory conditions. In contrast, tissues expressing exclusively Crb_A (epidermis, trachea, fore- and hindgut) secrete the rigid protective cuticle and form only irregular apical membrane folds during cuticle deposition (Uv and Moussian, 2010).

*crb^p17F5^* and *crb^p13A9^* mutants only develop one of the many described *crb* phenotypes – they undergo light-dependent PRC degeneration. Crb_C could not be detected in larval eye imaginal discs, but is only upregulated in the last 25% of pupal development, consistent with the absence of any morphological PRC phenotype in the mutants. Light-dependent retinal degeneration occurs in flies lacking all Crb isoforms (e.g. *crb^11A22^*) (Chartier et al., 2012; Johnson et al., 2002), raising the question whether it is the absence of Crb_C that causes retinal degeneration in *crb^11A22^* mutant PRCs. Since mutants affecting only Crb_A or Crb_B/D are not available, this question can presently not be answered. Interestingly, mutations in human *CRB1* result in RP12-asssociated blindness (den Hollander et al., 1999), despite the fact that *CRB2* and *CRB3* are also expressed in the retina (Lemmers et al., 2004; van den Hurk et al., 2005). This indicates that human CRB1 and *Drosophila* Crb_C have specific functions, which cannot be replaced by the other isoforms. Preliminary results indicate that PRCs of *crb^P13A9^* homozygous flies accumulate more intracellular Rhodopsin-carrying vesicles when exposed to light, a typical sign of retinal degeneration in flies (Pocha et al., 2011), making this allele an ideal source to further unravel the molecular mechanisms by which *Drosophila crb* protects photoreceptor cells from the detrimental consequences of light-induced cell stress.

Crb_C protein differs from Crb_A and Crb_B/D by the presence of additional 107 amino acids. This region adds one more EGF-like repeat and is highly conserved in all *Drosophila* species for which the genomic sequence has been annotated (https://genome.ucsc.edu/). EGF-like repeats are about 40 amino acids long, characterised by six conserved, regularly spaced cysteine residues, which allow the formation of three conserved disulphide bonds (Appella et al., 1988), and are found in secreted and transmembrane proteins of all metazoan. EGF-like repeats have been shown to mediate specific protein-protein interactions. In the Notch receptor of *Drosophila*, for example, EGF-like repeat #11 and #12 are sufficient for the interaction of Notch with its ligands Delta and Serrate, which mediates not only signalling but also adhesion (Rebay et al., 1991; Sakamoto et al., 2005). In the low-density lipoprotein receptor (LDLR), binding of EGF-like repeat A to PCSK9 (proprotein convertase subtilisin/kexin type 9) reduces the surface levels of the receptor and promotes its degradation (Zhang et al., 2007). One can speculate that the additional EGF-like repeat in Crb_C may modify the interaction with other proteins or may affect the homophilic interaction between the extracellular domains of Crb molecules, as suggested to occur in the *Drosophila* follicle epithelium (Letizia et al., 2013). Interestingly, many mutations in human *CRB1*, which are associated with RP12, are missense mutations in individual EGF-like repeats (Bujakowska et al., 2012). Whether these mutations result in structural changes of the EGF-like repeats, which may affect the global organisation and/or the stability of the extracellular region, or whether they affect a specific interaction of the respective EGF-like repeat with a binding partner, is not known.

Exon 5 also includes a stretch of amino acids rich in threonine residues, known substrates for mucin-type O-glycosylation. In fact, the difference in mobility of Crb_C in comparison to Crb_A under denaturing conditions may not only be due to the size difference, but also to an increased O-glycosylation. O-glycosylation has been documented as an essential protein modification highly conserved in evolution (Bennett et al., 2012; Tran and Ten Hagen, 2013). Various functions are associated with O-glycosylation in vertebrates, including the regulation of protein conformation, protein sorting and protein secretion. The embryonic epithelia that express Crb_C, e.g. the salivary gland, the hindgut and the Malpighian tubules, are also positive for several lectins, which detect O-linked glycans, particularly on the apical surface of these epithelia (Tian and Ten Hagen, 2007). In addition, these tissues express a number of genes encoding members of the PGANT (polypeptide *N*-acetylgalactosaminyltransferase) family (Tran et al., 2012). Whether any of these lectins detects Crb_C has to be further analysed. Beside mucin-type O-glycosylation, Crb and mouse Crb2 can be modified by EGF-specific O-glycosylation. Seven EGF-repeats of Crb are predicted to be O-glycosylated by the O-glycosyltransferase Rumi, the *Drosophila* homologue of mammalian POGLUT1. However, mutating all Rumi target sites did not recapitulate the *rumi* loss-of-function phenotype, which is characterised by fused rhabdomeres (Haltom et al., 2014). In contrast, loss of this modification in mouse Crb2 resulted in impaired apical localisation of Crb2 in epithelia of the developing mouse embryos and gastrulation arrest (Ramkumar et al., 2015).

Alternative splicing of pre-mRNA is a major mechanism used in higher eukaryotes to increase protein diversity. Data from the Ensembl annotation indicate that 45% of all *Drosophila* genes and at least 88% of all human genes encode transcripts undergoing alternative splicing (Lee and Rio, 2015; Pan et al., 2008). The region from exon 3 to exon 6 of *crb* reveals a high degree of conservation in all *Drosophila* species (but not in other insects such as *Apis mellifera* or *Tribolium castaneum*), and all exon/intron boundaries fit with the regular GU/AG splicing signal recognised by the major spliceosome. Exon 4 and 5 present in the *crb-RB/RD* and *crb-RC* mRNAs, respectively, are, based on the prediction, mutually exclusive exons, meaning that these exons never occur in the same mRNA. In the *D. melanogaster* exome, 1297 of the roughly 55,000 annotated exons are predicted to be mutually exclusive exons, but only 261 of these are predicted to be internal exons (Hatje and Kollmar, 2014). The most striking example of mutually exclusive exons is found in the *Drosophila* homologue of *Down syndrome adhesion molecule* (*Dscam*), which can potentially encode more than 38,000 protein variants by using mutually exclusive exons (Schmucker et al., 2000). Mutually exclusive splicing depends on several regulatory mechanisms, including cis-acting sequences on the pre-mRNA, trans-acting RNA-binding proteins and signalling molecules controlling these events (Fu and Ares, 2014; Lee and Rio, 2015; Pohl et al., 2013). Recent results suggested that the helicase Obelus controls the exclusion of exon 5 of *crb* at early embryonic stages. Absence of Obelus results in premature/ectopic expression of *crb-RC* mRNA during embryonic development, and induces polarity defects (Vichas et al., 2015). In the future, it may not only be interesting to elucidate tissue- and stage-specific regulators involved in the expression of this alternatively spliced mRNA, but also to unravel the role of the extracellular region and its difference between the isoforms.

## MATERIALS AND METHODS

### Fly stocks and genetics

Flies were kept at 25°C. The following stocks and mutant alleles were used: Oregon R and w* serving as controls, *sdt^K85^* [amorphic allele (Berger et al., 2007)], *crb^11A22^*, *crb^8F105^* and *crb^GX24^* [amorphic alleles (Huang et al., 2009; Jürgens et al., 1984; Wodarz et al., 1993)], *crb^p17F5^* and *crb^p13A9^* (this work; see below). *crb* mutant stocks were balanced over *twist*-GAL4, UAS-EGFP TM3 (Bloomington Stock Center). Eyes mosaic for *crb* were generated by crossing *yw eyFLP;;FRT82B w^+^ cl3R3/TM6B* males (Newsome et al., 2000) to *w;;FRT82B crb^11A22^/TM6B* females. All retinal analyses were performed using age-matched female flies. Light-induced retinal degeneration was analysed as published (Johnson et al., 2002). For life span analyses, virgin adults (within 6 h after eclosion) were collected and maintained in groups of 15-20 adults. Flies were placed at 29°C and transferred on fresh food twice a week. The number of surviving adults was recorded with each transfer until all the flies in the vial had died.

### Generation of *crb_C* isoform-specific mutant fly lines

To isolate point mutations in the *crb* locus (FlyBase ID FBgn0259685) that specifically affect isoform Crb_C, we screened a library of 2400 fly lines that contained isogenised third chromosomes, which potentially carry point mutations caused by EMS treatment. Our approach included the alternatively spliced exon 5, thus specifically targeting the *crb_c* mRNA encoding region (RefSeq ID NM_001260355). We amplified exon 4 and 5 from genomic DNA making use of a nested PCR approach (outer primer: forward GATCATTTGTTGGGTACTGC, reverse TAAACCCAAGAACCACAAGG, inner primer: forward TGTAAAACGACGGCCAGTGGTCTTAATTTCTCCACCTAACC, reverse AGGAAACAGCTATGACCATGTGGATCATCAGTGACATGC). All PCR reactions were performed in 10 μl volume with an annealing temperature of 57°C in 384-well format making use of automated liquid handling tools. PCR fragments were sequenced by Sanger sequencing using the M13 reverse primer AGGAAACAGCTATGACCAT and screened for point mutations with the PolyPhred Software tool (Stephens et al., 2006; Winkler et al., 2011). All primary hits that were predicted as potentially deleterious mutations upon translation were verified in an independent PCR amplification and Sanger sequencing reaction.

### Production of α-Crb antibodies

Antisera and monoclonal antibodies against the amino acids encoded by the common exon 3 were obtained by repeated immunisation of rats using a His-fusion protein containing amino acids 195-525:

ANSTYNSQFLTNQDIGYKDAILILGNSFSGCLLDGPGLQFVNNSTVQNVVFGHCPLTPGPCSDHDLFTRLPDNFCLNDPCMGHGTCSSSPEGYECRCTARYSGKNCQKDNGSPCAKNPCENGGSCLENSRGDYQCFCDPNHSGQHCETEVNIHPLCQTNPCLNNGACVVIGGSGALTCECPKGYAGARCEVDTDECASQPCQNNGSCIDRINGFSCDCSGTGYTGAFCQTNVDECDKNPCLNGGRCFDTYGWYTCQCLDGWGGEICDRPMTCQTQQCLNGGTCLDKPIGFQCLCPPEYTGELCQIAPSCAQQCPIDSECVGG KCVCKPGSS (Antibody Facility, Max-Planck Institute). This antibody detects all Crb isoforms.

Antisera against the amino acid sequence encoded by exon 4 and specific for Crb_B/D were obtained by repeated immunisation of rats using a His-fusion protein containing the amino acid sequence encoded by exon 4 specific for Crb_B/D: SDMEPLTPLELDILDATLCPSEKKKRYISPEWLKRKRCELKL.

Antisera against Crb_C were obtained by repeated immunisation of rats using a His-fusion protein containing part of the amino acid sequence encoded by exon 5: KIPPITTSRTLVGTTTGSRRPPQQPLQSPTQRSASLNACPQENCLNGGTCLGYSGNYSCICASGYTGLAYICPGLS. The antibody from rat 1 was used for immunohistochemistry, and the one from rat 3 for western blot experiments.

### Whole-mount immunohistochemistry of *Drosophila* embryos, imaginal discs and eyes

Antibody staining of embryos, imaginal discs and adult retina were essentially performed as previously described (Gurudev et al., 2014; Klose et al., 2013; Lin et al., 2015; Muschalik and Knust, 2011). For staging pupae, white prepupae (0 h APF) were collected and aged (72 h APF at 25°C). The following primary antibodies were used: rat α-Crb2.8 (1:1000; Richard et al., 2006), mouse monoclonal α-Crb_Cq4 (1:1000; Tepass et al., 1990), rat α-Crb_Ex3, detecting the common exon 3 (1:200; this work), rat α-Crb_intra2662 raised against the cytoplasmic tail (1:100; Kumichel et al., 2015), rat α-Crb_B/D (1:200; this work), rat α-Crb_C (1:200; this work), rabbit α-Krüppel (1:500; kindly provided by H. Jäckle, MPI for Biophysical Chemistry, Göttingen, Germany; Gaul et al., 1987), rabbit α-Sox100B (1:25,000; kindly provided by S. Russell, Department of Genetics, University of Cambridge, Cambridge, UK; Hui Yong Loh and Russell, 2000), mouse α-Chaoptin (1:25, mAB24B10, DSHB). F-actin was visualised with Alexa-Fluor-488–phalloidin (1:40; Invitrogen). Images were taken on a Zeiss LSM 510 or Olympus FV100 and processed using ImageJ/Fiji, Adobe Photoshop CS3 & CS5.1 and Adobe Illustrator CS3 for image assembly.

### Transmission electron microscopy and quantification of stalk membrane length

Fixation of adult eyes and ultra-thin sections for transmission electron microscopy was performed as described (Mishra and Knust, 2013). Sections were contrasted and analysed using a FEI Tecnai 12 Bio Twin. For quantitative analysis of the stalk membrane length, images were taken at a magnification of 56,000 using a TemCam F2114A digital camera. The stalk membranes of nine ommatidia, obtained from eyes of three different flies, were measured for each genotype using ImageJ. Difference in stalk length between genotypes was assessed by ANOVA followed by post hoc Bonferroni Test using OriginLab. Graphs were drawn using Microsoft Excel.

### Overexpression in *Drosophila* Schneider cells S2R^+^ and western blot analyses

Overexpression in S2R^+^ cells, preparation of head lysates and western blots were essentially performed as previously described (Kumichel et al., 2015; Pocha et al., 2011). pUAST-plasmids used were as follows. The Crb_A encoding plasmid has been described (Wodarz et al., 1995). For generation of pUAST-*crb_C*, part of the *crb_C* mRNA was amplified by RT-PCR using the following primers: SmaI_f: GTGGTCTTTGGTCACTGTCC, NsiI_r: CAAATACAGGAATAATTGCCAC. The PCR fragment was cloned into *pBS_p30.1*, which contains the *crb_A* minigene (Wodarz et al., 1995) (kindly provided by N. Muschalik, Knust Lab), resulting in a *crb_C* minigene that was further cloned into the pUASTattB vector (Bischof et al., 2007). As control for overexpression, pAct5C-GAL4 was transfected alone. N- and O-deglycosylation was performed using the protein deglycosylation mix (New England Biolabs) as suggested by the manufacturer, except that proteins were denatured at 65°C for 15 min. Immunodetection was done with rabbit α-Crb_intra2662, rat α-Crb2.8 (0.5 μg/ml; Kumichel et al., 2015), or rat α-Crb_C antibodies (0.5 μg/ml) as primary and goat α-rabbit and α-rat antibodies conjugated with peroxidase (1:10,000; Sigma-Aldrich) as secondary antibodies using the Amersham ECL Western Blotting Detection Reagent (GE Healthcare Life Sciences).

### *In silico* sequence analysis

The molecular weight was calculated with Compute pI/Mw tool (http://web.expasy.org/compute_pi/; Gasteiger et al., 2005). Glycosylation was predicted with NetNglyc (http://www.cbs.dtu.dk/services/NetNGlyc/) and NetOglyc 3.1 (http://www.cbs.dtu.dk/services/NetOGlyc-3.1/); (Julenius et al., 2005).

### RT-PCR experiments and Real-Time quantitative RT-PCR (qRT-PCR)

The large intestine was dissected from wandering wild-type third instar larvae in ice-cold PBS. mRNA was isolated using the μMACS™ mRNA Isolation Kit (Miltenyi Biotec GmbH, Bergisch Gladbach, Germany) and 0.8 U/μl RNaseOUT™ (Invitrogen) was added. Subsequently, cDNA synthesis was performed using the SuperScript^®^ III Reverse Transcriptase Kit (Invitrogen) with the reverse primer CGCATATGTGACATCGACAT. For PCR reactions, the primer pairs 1 and 2 listed in Table S2 were used. PCR was carried out on an Eppendorf Mastercycler ep gradient S with the following program: step 1: 15 min 98°C; step 2: 20 s 98°C; step 3: 20 s 55°C for primer pair 1 or 53°C for primer pair 2; step 4: 1 min 20 s 72°C; step 5: 10 min 72°C. Steps 2-4 were repeated 36 times. A 20 μl PCR mix consisted of 10 μl 2x HotStarTaq Master Mix (QIAGEN), 1 μl 10 μM forward primer, 1 μl 10 μM reverse primer, 1 μl cDNA, 7 μl dH_2_O. PCR products were analysed by agarose gel electrophoresis.

RNA extraction from heads was carried out using a standard Trizol/chloroform-based extraction with ethanol purification. Approximately 10 pupal heads or 10 adult heads of Oregon R per sample constituted one biological replicate and each experiment included two such replicates. cDNA generation was carried out using SuperScript™ First Strand Synthesis System (Invitrogen) with a starting amount of 1 μg total RNA. Primers were designed using Primer-Blast, NCBI (Ye et al., 2012) and are listed in Table S2. Triplicate cDNA aliquots for each sample served as templates for RT-qPCR using ABsolute qPCR SYBR Green Mix (Thermo Fisher Scientific) on a Stratagene Mx3000P qPCR (Thermo Fisher Scientific) system. Fold-change was calculated after normalization to the housekeeping gene *Gapdh1* using the Δ Δ *Ct* method.

### RNA sequencing

Total RNA from heads and eyes of Oregon R (wild-type) female flies was extracted using routine Trizol/chloroform based extraction with ethanol purification. Three biological replicates constituting of 25 heads or 50 dissected eyes were subjected to RNA extraction and analyses. mRNA isolation by poly-A enrichment, strand-specific RNA-Seq library preparation and sequencing was carried out by the Deep Sequencing Group SFB 655 at Biotechnology Center, TU Dresden with 75-bp single read sequencing on the Illumina HiSeq 2500. A total of 30 million reads per sample were obtained. Sequence analysis was carried out by the Scientific Computing Facility (MPI-CBG) by mapping to the *Drosophila* genome [Genome assembly: Ensembl BDGP6 (GCA_000001215.4)] using the RNA-Seq aligner STAR (v_2.3.1z). Transcript expression was quantified using Cuffdiff method (Trapnell et al., 2013) as fragments per kilobase of exon per million fragments mapped (FPKM). Abundance values for transcripts between heads and eyes were compared for significant differences using Mann–Whitney–Wilcoxon test.

## References

[BIO020040C1] Alves C. H., Pellissier L. P., Vos R. M., Garcia Garrido M., Sothilingam V., Seide C., Beck S. C., Klooster J., Furukawa T., Flannery J. G. et al. (2014). Targeted ablation of Crb2 in photoreceptor cells induces retinitis pigmentosa. *Hum. Mol. Genet.* 23, 3384-3401. 10.1093/hmg/ddu048 24493795

[BIO020040C2] Appella E., Weber I. T. and Blasi F. (1988). Structure and function of epidermal growth factor-like regions in proteins. *FEBS Lett.* 231, 1-4. 10.1016/0014-5793(88)80690-2 3282918

[BIO020040C3] Bachmann A., Schneider M., Grawe F., Theilenberg E. and Knust E. (2001). *Drosophila* Stardust is a partner of Crumbs in the control of epithelial cell polarity. *Nature* 414, 638-643. 10.1038/414638a 11740560

[BIO020040C90] Bazellieres E., Assemat E., Arsanto J. P., Le Bivic A. and Massey-Harroche D. (2009). Crumbs proteins in epithelial morphogenesis. *Front. Biosci.* 14, 2149-2169. 10.2741/3368 19273190

[BIO020040C4] Bennett E. P., Mandel U., Clausen H., Gerken T. A., Fritz T. A. and Tabak L. A. (2012). Control of mucin-type O-glycosylation: a classification of the polypeptide GalNAc-transferase gene family. *Glycobiology* 22, 736-756. 10.1093/glycob/cwr182 22183981 PMC3409716

[BIO020040C5] Berger S., Bulgakova N. A., Grawe F., Johnson K. and Knust E. (2007). Unraveling the genetic complexity of *Drosophila stardust* during photoreceptor morphogenesis and prevention of light-induced degeneration. *Genetics* 176, 2189-2200. 10.1534/genetics.107.071449 17603117 PMC1950624

[BIO020040C6] Bischof J., Maeda R. K., Hediger M., Karch F. and Basler K. (2007). An optimized transgenesis system for *Drosophila* using germ-line-specific phiC31 integrases. *Proc. Natl. Acad. Sci. USA* 104, 3312-3317. 10.1073/pnas.0611511104 17360644 PMC1805588

[BIO020040C7] Boggiano J. C. and Fehon R. G. (2012). Growth control by committee: intercellular junctions, cell polarity, and the cytoskeleton regulate Hippo signaling. *Dev. Cell* 22, 695-702. 10.1016/j.devcel.2012.03.013 22516196 PMC3376383

[BIO020040C8] Bujakowska K., Audo I., Mohand-Saïd S., Lancelot M.-E., Antonio A., Germain A., Léveillard T., Letexier M., Saraiva J.-P., Lonjou C. et al. (2012). CRB1 mutations in inherited retinal dystrophies. *Hum. Mutat.* 33, 306-315. 10.1002/humu.21653 22065545 PMC3293109

[BIO020040C9] Bulgakova N. A. and Knust E. (2009). The Crumbs complex: from epithelial-cell polarity to retinal degeneration. *J. Cell Sci.* 122, 2587-2596. 10.1242/jcs.023648 19625503

[BIO020040C10] Campbell K., Knust E. and Skaer H. (2009). Crumbs stabilises epithelial polarity during tissue remodelling. *J. Cell Sci.* 122, 2604-2612. 10.1242/jcs.047183 19567473 PMC2909315

[BIO020040C11] Charrier L. E., Loie E. and Laprise P. (2015). Mouse Crumbs3 sustains epithelial tissue morphogenesis in vivo. *Sci. Rep.* 5, 17699 10.1038/srep17699 26631503 PMC4668553

[BIO020040C12] Chartier F. J.-M., Hardy J.-L. and Laprise P. (2012). Crumbs limits oxidase-dependent signaling to maintain epithelial integrity and prevent photoreceptor cell death. *J. Cell Biol.* 198, 991-998. 10.1083/jcb.201203083 22965909 PMC3444775

[BIO020040C13] Chen C.-L., Gajewski K. M., Hamaratoglu F., Bossuyt W., Sansores-Garcia L., Tao C. and Halder G. (2010). The apical-basal cell polarity determinant Crumbs regulates Hippo signaling in *Drosophila*. *Proc. Natl. Acad. Sci. USA* 107, 15810-15815. 10.1073/pnas.1004060107 20798049 PMC2936591

[BIO020040C14] den Hollander A. I., ten Brink J. B., de Kok Y. J. M., van Soest S., van den Born L. I., van Driel M. A., van de Pol D. J. R., Payne A. M., Bhattacharya S. S., Kellner U. et al. (1999). Mutations in a human homologue of *Drosophila crumbs* cause retinitis pigmentosa (RP12). *Nat. Genet.* 23, 217-221. 10.1038/13848 10508521

[BIO020040C15] den Hollander A. I., Johnson K., de Kok Y. J. M., Klebes A., Brunner H. G., Knust E. and Cremers F. P. M. (2001). CRB1 has a cytoplasmic domain that is functionally conserved between human and *Drosophila*. *Hum. Mol. Genet.* 10, 2767-2773. 10.1093/hmg/10.24.2767 11734541

[BIO020040C16] Ebarasi L., Ashraf S., Bierzynska A., Gee H. Y., McCarthy H. J., Lovric S., Sadowski C. E., Pabst W., Vega-Warner V., Fang H. et al. (2015). Defects of CRB2 cause steroid-resistant nephrotic syndrome. *Am. J. Hum. Genet.* 96, 153-161. 10.1016/j.ajhg.2014.11.014 25557779 PMC4289689

[BIO020040C17] Flores-Benitez D. and Knust E. (2015). Crumbs is an essential regulator of cytoskeletal dynamics and cell-cell adhesion during dorsal closure in *Drosophila*. *Elife* 4, e07398 10.7554/eLife.07398 26544546 PMC4718732

[BIO020040C18] Flores-Benitez D. and Knust E. (2016). Dynamics of epithelial cell polarity in *Drosophila*: how to regulate the regulators? *Curr. Opin. Cell Biol.* 42, 13-21. 10.1016/j.ceb.2016.03.018 27085003

[BIO020040C19] Fu X.-D. and Ares M. Jr (2014). Context-dependent control of alternative splicing by RNA-binding proteins. *Nat. Rev. Genet.* 15, 689-701. 10.1038/nrg3778 25112293 PMC4440546

[BIO020040C20] Fuss B. and Hoch M. (1998). *Drosophila* endoderm development requires a novel homeobox gene which is a target of Wingless and Dpp signalling. *Mech. Dev.* 79, 83-97. 10.1016/S0925-4773(98)00172-5 10349623

[BIO020040C21] Gaul U., Seifert E., Schuh R. and Jäckle H. (1987). Analysis of Krüppel protein distribution during early *Drosophila* development reveals posttranscriptional regulation. *Cell* 50, 639-647. 10.1016/0092-8674(87)90037-7 3111719

[BIO020040C91] Gasteiger E., Hoogland C., Gattiker A., Duvaud S., Wilkins M. R., Appel R. D. and Bairoch A. (2005). Protein Identification and Analysis Tools on the ExPASy Server. In *The Proteomics Protocols Handbook* (ed. Walker J. M.). Totowa, NJ: Humana Press.

[BIO020040C22] Genevet A. and Tapon N. (2011). The Hippo pathway and apico-basal cell polarity. *Biochem. J.* 436, 213-224. 10.1042/BJ20110217 21568941

[BIO020040C23] Grawe F., Wodarz A., Lee B., Knust E. and Skaer H. (1996). The *Drosophila* genes *crumbs* and *stardust* are involved in the biogenesis of adherens junctions. *Development* 122, 951-959. 8631272 10.1242/dev.122.3.951

[BIO020040C24] Gurudev N., Yuan M. and Knust E. (2014). *chaoptin*, *prominin*, *eyes shut* and *crumbs* form a genetic network controlling the apical compartment of *Drosophila* photoreceptor cells. *Biol. Open* 3, 332-341. 10.1242/bio.20147310 24705015 PMC4021355

[BIO020040C25] Hafezi Y., Bosch J. A. and Hariharan I. K. (2012). Differences in levels of the transmembrane protein Crumbs can influence cell survival at clonal boundaries. *Dev. Biol.* 368, 358-369. 10.1016/j.ydbio.2012.06.001 22683826 PMC3412113

[BIO020040C26] Haltom A. R., Lee T. V., Harvey B. M., Leonardi J., Chen Y.-J., Hong Y., Haltiwanger R. S. and Jafar-Nejad H. (2014). The protein O-glucosyltransferase Rumi modifies eyes shut to promote rhabdomere separation in *Drosophila*. *PLoS Genet.* 10, e1004795 10.1371/journal.pgen.1004795 25412384 PMC4238978

[BIO020040C27] Hatje K. and Kollmar M. (2014). Kassiopeia: a database and web application for the analysis of mutually exclusive exomes of eukaryotes. *BMC Genomics* 15, 115 10.1186/1471-2164-15-115 24507667 PMC3923563

[BIO020040C28] Herranz H., Stamataki E., Feiguin F. and Milán M. (2006). Self-refinement of Notch activity through the transmembrane protein Crumbs: modulation of γ-secretase activity. *EMBO Rep.* 7, 297-302. 10.1038/sj.embor.7400617 16440003 PMC1456882

[BIO020040C29] Hong Y., Stronach B., Perrimon N., Jan L. Y. and Jan Y. N. (2001). *Drosophila* Stardust interacts with Crumbs to control polarity of epithelia but not neuroblasts. *Nature* 414, 634-638. 10.1038/414634a 11740559

[BIO020040C30] Huang J., Zhou W., Dong W., Watson A. M. and Hong Y. (2009). Directed, efficient, and versatile modifications of the *Drosophila* genome by genomic engineering. *Proc. Natl. Acad. Sci. USA* 106, 8284-8289. 10.1073/pnas.0900641106 19429710 PMC2688891

[BIO020040C31] Hui Yong Loh S. and Russell S. (2000). A *Drosophila* group E Sox gene is dynamically expressed in the embryonic alimentary canal. *Mech. Dev.* 93, 185-188. 10.1016/S0925-4773(00)00258-6 10781954

[BIO020040C32] Iwaki D. D. and Lengyel J. A. (2002). A Delta-Notch signaling border regulated by Engrailed/Invected repression specifies boundary cells in the *Drosophila* hindgut. *Mech. Dev.* 114, 71-84. 10.1016/S0925-4773(02)00061-8 12175491

[BIO020040C33] Izaddoost S., Nam S.-C., Bhat M. A., Bellen H. J. and Choi K.-W. (2002). *Drosophila* Crumbs is a positional cue in photoreceptor adherens junctions and rhabdomeres. *Nature* 416, 178-183. 10.1038/nature720 11850624

[BIO020040C34] Johnson K., Grawe F., Grzeschik N. and Knust E. (2002). *Drosophila* Crumbs is required to inhibit light-induced photoreceptor degeneration. *Curr. Biol.* 12, 1675-1680. 10.1016/S0960-9822(02)01180-6 12361571

[BIO020040C35] Julenius K., Molgaard A., Gupta R. and Brunak S. (2005). Prediction, conservation analysis, and structural characterization of mammalian mucin-type O-glycosylation sites. *Glycobiology* 15, 153-164. 10.1093/glycob/cwh151 15385431

[BIO020040C36] Jürgens G., Wieschaus E., Nüsslein-Volhard C. and Kluding H. (1984). Mutations affecting the pattern of the larval cuticle in *Drosophila melanogaster*. II. Zygotic loci on the third chromosome. *Roux's Arch. Dev. Biol.* 193, 283-295. 10.1007/BF00848157 28305338

[BIO020040C37] Kilic A., Klose S., Dobberstein B., Knust E. and Kapp K. (2010). The *Drosophila* Crumbs signal peptide is unusually long and is a substrate for signal peptide peptidase. *Eur. J. Cell Biol.* 89, 449-461. 10.1016/j.ejcb.2010.02.001 20189678

[BIO020040C38] Klebes A. and Knust E. (2000). A conserved motif in Crumbs is required for E-cadherin localisation and zonula adherens formation in *Drosophila*. *Curr. Biol.* 10, 76-85. 10.1016/S0960-9822(99)00277-8 10662667

[BIO020040C39] Klose S., Flores-Benitez D., Riedel F. and Knust E. (2013). Fosmid-based structure-function analysis reveals functionally distinct domains in the cytoplasmic domain of *Drosophila* Crumbs. *G3* 3, 153-165. 10.1534/g3.112.005074 23390593 PMC3564977

[BIO020040C40] Kumichel A. and Knust E. (2014). Apical localisation of Crumbs in the boundary cells of the *Drosophila* hindgut is independent of its canonical interaction partner Stardust. *PLoS ONE* 9, e94038 10.1371/journal.pone.0094038 24710316 PMC3977972

[BIO020040C41] Kumichel A., Kapp K. and Knust E. (2015). A conserved di-basic motif of *Drosophila* crumbs contributes to efficient ER export. *Traffic* 16, 604-616. 10.1111/tra.12273 25753515 PMC6681134

[BIO020040C42] Laprise P., Beronja S., Silva-Gagliardi N. F., Pellikka M., Jensen A. M., McGlade C. J. and Tepass U. (2006). The FERM protein Yurt is a negative regulatory component of the Crumbs complex that controls epithelial polarity and apical membrane size. *Dev. Cell* 11, 363-374. 10.1016/j.devcel.2006.06.001 16950127 PMC2834949

[BIO020040C43] Le Bivic A. (2013). Evolution and cell physiology. 4. Why invent yet another protein complex to build junctions in epithelial cells? *Am. J. Physiol. Cell Physiol.* 305, C1193-C1201. 10.1152/ajpcell.00272.2013 24025867

[BIO020040C44] Lee Y. and Rio D. C. (2015). Mechanisms and regulation of alternative pre-mRNA splicing. *Annu. Rev. Biochem.* 84, 291-323. 10.1146/annurev-biochem-060614-034316 25784052 PMC4526142

[BIO020040C45] Lemmers C., Michel D., Lane-Guermonprez L., Delgrossi M.-H., Médina E., Arsanto J.-P. and Le Bivic A. (2004). CRB3 binds directly to Par6 and regulates the morphogenesis of the tight junctions in mammalian epithelial cells. *Mol. Biol. Cell* 15, 1324-1333. 10.1091/mbc.E03-04-0235 14718572 PMC363137

[BIO020040C46] Letizia A., Ricardo S., Moussian B., Martin N. and Llimargas M. (2013). A functional role of the extracellular domain of Crumbs in cell architecture and apicobasal polarity. *J. Cell Sci.* 126, 2157-2163. 10.1242/jcs.122382 23525000

[BIO020040C47] Lin Y.-H., Currinn H., Pocha S. M., Rothnie A., Wassmer T. and Knust E. (2015). AP-2-complex-mediated endocytosis of *Drosophila Crumbs* regulates polarity by antagonizing Stardust. *J. Cell Sci.* 128, 4538-4549. 10.1242/jcs.174573 26527400

[BIO020040C48] Ling C., Zheng Y., Yin F., Yu J., Huang J., Hong Y., Wu S. and Pan D. (2010). The apical transmembrane protein Crumbs functions as a tumor suppressor that regulates Hippo signaling by binding to Expanded. *Proc. Natl. Acad. Sci. USA* 107, 10532-10537. 10.1073/pnas.1004279107 20498073 PMC2890787

[BIO020040C49] Makarova O., Roh M. H., Liu C.-J., Laurinec S. and Margolis B. (2003). Mammalian Crumbs3 is a small transmembrane protein linked to protein associated with Lin-7 (Pals1). *Gene* 302, 21-29. 10.1016/S0378111902010843 12527193

[BIO020040C50] Mishra M. and Knust E. (2013). Analysis of the *Drosophila* compound eye with light and electron microscopy. *Methods Mol. Biol.* 935, 161-182. 10.1007/978-1-62703-080-9_11 23150367

[BIO020040C51] Muschalik N. and Knust E. (2011). Increased levels of the cytoplasmic domain of Crumbs repolarise developing *Drosophila* photoreceptors. *J. Cell Sci.* 124, 3715-3725. 10.1242/jcs.091223 22025631

[BIO020040C52] Nemetschke L. and Knust E. (2016). *Drosophila* Crumbs prevents ectopic Notch activation in developing wings by inhibiting ligand-independent endocytosis. *Development* 143, 4543-4553. 10.1242/dev.141762 27899511

[BIO020040C53] Newsome T. P., Asling B. and Dickson B. J. (2000). Analysis of *Drosophila* photoreceptor axon guidance in eye-specific mosaics. *Development* 127, 851-860. 10648243 10.1242/dev.127.4.851

[BIO020040C54] Pan Q., Shai O., Lee L. J., Frey B. J. and Blencowe B. J. (2008). Deep surveying of alternative splicing complexity in the human transcriptome by high-throughput sequencing. *Nat. Genet.* 40, 1413-1415. 10.1038/ng.259 18978789

[BIO020040C55] Pellikka M., Tanentzapf G., Pinto M., Smith C., McGlade C. J., Ready D. F. and Tepass U. (2002). Crumbs, the *Drosophila* homologue of human CRB1/RP12, is essential for photoreceptor morphogenesis. *Nature* 416, 143-149. 10.1038/nature721 11850625

[BIO020040C56] Pocha S. M., Shevchenko A. and Knust E. (2011). Crumbs regulates rhodopsin transport by interacting with and stabilizing myosin V. *J. Cell Biol.* 195, 827-838. 10.1083/jcb.201105144 22105348 PMC3257572

[BIO020040C57] Pohl M., Bortfeldt R. H., Grützmann K. and Schuster S. (2013). Alternative splicing of mutually exclusive exons - a review. *Biosystems* 114, 31-38. 10.1016/j.biosystems.2013.07.003 23850531

[BIO020040C58] Ramkumar N., Harvey B. M., Lee J. D., Alcorn H. L., Silva-Gagliardi N. F., McGlade C. J., Bestor T. H., Wijnholds J., Haltiwanger R. S. and Anderson K. V. (2015). Protein O-Glucosyltransferase 1 (POGLUT1) promotes mouse gastrulation through modification of the apical polarity protein CRUMBS2. *PLoS Genet.* 11, e1005551 10.1371/journal.pgen.1005551 26496195 PMC4619674

[BIO020040C59] Rebay I., Fleming R. J., Fehon R. G., Cherbas L., Cherbas P. and Artavanis-Tsakonas S. (1991). Specific EGF repeats of Notch mediate interactions with Delta and Serrate: implications for Notch as a multifunctional receptor. *Cell* 67, 687-699. 10.1016/0092-8674(91)90064-6 1657403

[BIO020040C60] Richard M., Grawe F. and Knust E. (2006). *D*PATJ plays a role in retinal morphogenesis and protects against light-dependent degeneration of photoreceptor cells in the *Drosophila* eye. *Dev. Dyn.* 235, 895-907. 10.1002/dvdy.20595 16245332

[BIO020040C61] Richard M., Muschalik N., Grawe F., Özüyaman S. and Knust E. (2009). A role for the extracellular domain of Crumbs in morphogenesis of *Drosophila* photoreceptor cells. *Eur. J. Cell Biol.* 88, 765-777. 10.1016/j.ejcb.2009.07.006 19717208

[BIO020040C62] Richardson E. C. N. and Pichaud F. (2010). Crumbs is required to achieve proper organ size control during Drosophila head development. *Development* 137, 641-650. 10.1242/dev.041913 20110329 PMC2827617

[BIO020040C63] Robinson B. S., Huang J., Hong Y. and Moberg K. H. (2010). Crumbs regulates Salvador/Warts/Hippo signaling in *Drosophila* via the FERM-domain protein expanded. *Curr. Biol.* 20, 582-590. 10.1016/j.cub.2010.03.019 20362445 PMC2855393

[BIO020040C64] Roh M. H., Fan S., Liu C.-J. and Margolis B. (2003). The Crumbs3-Pals1 complex participates in the establishment of polarity in mammalian epithelial cells. *J. Cell Sci.* 116, 2895-2906. 10.1242/jcs.00500 12771187

[BIO020040C65] Sakamoto K., Chao W. S., Katsube K.-I. and Yamaguchi A. (2005). Distinct roles of EGF repeats for the Notch signaling system. *Exp. Cell Res.* 302, 281-291. 10.1016/j.yexcr.2004.09.016 15561108

[BIO020040C66] Schmucker D., Clemens J. C., Shu H., Worby C. A., Xiao J., Muda M., Dixon J. E. and Zipursky S. L. (2000). *Drosophila* Dscam is an axon guidance receptor exhibiting extraordinary molecular diversity. *Cell* 101, 671-684. 10.1016/S0092-8674(00)80878-8 10892653

[BIO020040C67] Slavotinek A., Kaylor J., Pierce H., Cahr M., DeWard S. J., Schneidman-Duhovny D., Alsadah A., Salem F., Schmajuk G. and Mehta L. (2015). CRB2 mutations produce a phenotype resembling congenital nephrosis, Finnish type, with cerebral ventriculomegaly and raised alpha-fetoprotein. *Am. J. Hum. Genet.* 96, 162-169. 10.1016/j.ajhg.2014.11.013 25557780 PMC4289687

[BIO020040C68] Stephens M., Sloan J. S., Robertson P. D., Scheet P. and Nickerson D. A. (2006). Automating sequence-based detection and genotyping of SNPs from diploid samples. *Nat. Genet.* 38, 375-381. 10.1038/ng1746 16493422

[BIO020040C69] Sun S. and Irvine K. D. (2016). Cellular organization and cytoskeletal regulation of the Hippo Signaling Network. *Trends Cell Biol.* 26, 694-704. 10.1016/j.tcb.2016.05.003 27268910 PMC4993636

[BIO020040C70] Szymaniak A. D., Mahoney J. E., Cardoso W. V. and Varelas X. (2015). Crumbs3-mediated polarity directs airway epithelial cell fate through the hippo pathway effector Yap. *Dev. Cell* 34, 283-296. 10.1016/j.devcel.2015.06.020 26235047 PMC4536126

[BIO020040C71] Tepass U. (1996). Crumbs, a component of the apical membrane, is required for zonula adherens formation in primary epithelia of *Drosophila*. *Dev. Biol.* 177, 217-225. 10.1006/dbio.1996.0157 8660889

[BIO020040C72] Tepass U. (2012). The apical polarity protein network in *Drosophila* epithelial cells: regulation of polarity, junctions, morphogenesis, cell growth, and survival. *Annu. Rev. Cell Dev. Biol.* 28, 655-685. 10.1146/annurev-cellbio-092910-154033 22881460

[BIO020040C73] Tepass U. and Knust E. (1990). Phenotypic and developmental analysis of mutations at the *crumbs* locus, a gene required for the development of epithelia in *Drosophila melanogaster*. *Roux's Arch. Dev. Biol.* 199, 189-206. 10.1007/BF01682078 28306104

[BIO020040C74] Tepass U. and Knust E. (1993). *crumbs* and *stardust* act in a genetic pathway that controls the organization of epithelia in *Drosophila melanogaster*. *Dev. Biol.* 159, 311-326. 10.1006/dbio.1993.1243 8365569

[BIO020040C75] Tepass U., Theres C. and Knust E. (1990). *crumbs* encodes an EGF-like protein expressed on apical membranes of *Drosophila* epithelial cells and required for organization of epithelia. *Cell* 61, 787-799. 10.1016/0092-8674(90)90189-L 2344615

[BIO020040C76] Tian E. and Ten Hagen K. G. (2007). O-linked glycan expression during *Drosophila* development. *Glycobiology* 17, 820-827. 10.1093/glycob/cwm056 17522109

[BIO020040C77] Tran D. T. and Ten Hagen K. G. (2013). Mucin-type O-glycosylation during development. *J. Biol. Chem.* 288, 6921-6929. 10.1074/jbc.R112.418558 23329828 PMC3591602

[BIO020040C78] Tran D. T., Zhang L., Zhang Y., Tian E., Earl L. A. and Ten Hagen K. G. (2012). Multiple members of the UDP-GalNAc: polypeptide N-acetylgalactosaminyltransferase family are essential for viability in *Drosophila*. *J. Biol. Chem.* 287, 5243-5252. 10.1074/jbc.M111.306159 22157008 PMC3285305

[BIO020040C79] Trapnell C., Hendrickson D. G., Sauvageau M., Goff L., Rinn J. L. and Pachter L. (2013). Differential analysis of gene regulation at transcript resolution with RNA-seq. *Nat. Biotech.* 31, 46-53. 10.1038/nbt.2450 PMC3869392 23222703

[BIO020040C80] Uv A. and Moussian B. (2010). The apical plasma membrane of *Drosophila* embryonic epithelia. *Eur. J. Cell Biol.* 89, 208-211. 10.1016/j.ejcb.2009.11.009 19944479

[BIO020040C81] van den Hurk J. A., Rashbass P., Roepman R., Davis J., Voesenek K. E., Arends M. L., Zonneveld M. N., van Roekel M. H., Cameron K., Rohrschneider K. et al. (2005). Characterization of the Crumbs homolog 2 (CRB2) gene and analysis of its role in retinitis pigmentosa and Leber congenital amaurosis. *Mol. Vis.* 11, 263-273. 15851977

[BIO020040C82] Vichas A., Laurie M. T. and Zallen J. A. (2015). The Ski2-family helicase Obelus regulates Crumbs alternative splicing and cell polarity. *J. Cell Biol.* 211, 1011-1024. 10.1083/jcb.201504083 26644515 PMC4674277

[BIO020040C83] Whiteman E. L., Fan S., Harder J. L., Walton K. D., Liu C.-J., Soofi A., Fogg V. C., Hershenson M. B., Dressler G. R., Deutsch G. H. et al. (2014). Crumbs3 is essential for proper epithelial development and viability. *Mol. Cell. Biol.* 34, 43-56. 10.1128/MCB.00999-13 24164893 PMC3911272

[BIO020040C84] Winkler S., Gscheidel N. and Brand M. (2011). Mutant generation in vertebrate model organisms by TILLING. *Methods Mol. Biol.* 770, 475-504. 10.1007/978-1-61779-210-6_19 21805277

[BIO020040C85] Wodarz A., Grawe F. and Knust E. (1993). Crumbs is involved in the control of apical protein targeting during *Drosophila* epithelial development. *Mech. Dev.* 44, 175-187. 10.1016/0925-4773(93)90066-7 8155580

[BIO020040C86] Wodarz A., Hinz U., Engelbert M. and Knust E. (1995). Expression of Crumbs confers apical character on plasma membrane domains of ectodermal epithelia of *Drosophila*. *Cell* 82, 67-76. 10.1016/0092-8674(95)90053-5 7606787

[BIO020040C87] Xiao Z., Patrakka J., Nukui M., Chi L., Niu D., Betsholtz C., Pikkarainen T., Vainio S. and Tryggvason K. (2011). Deficiency in Crumbs homolog 2 (Crb2) affects gastrulation and results in embryonic lethality in mice. *Dev. Dyn.* 240, 2646-2656. 10.1002/dvdy.22778 22072575

[BIO020040C88] Ye J., Coulouris G., Zaretskaya I., Cutcutache I., Rozen S. and Madden T. L. (2012). Primer-BLAST: a tool to design target-specific primers for polymerase chain reaction. *BMC Bioinformatics* 13, 134 10.1186/1471-2105-13-134 22708584 PMC3412702

[BIO020040C89] Zhang D.-W., Lagace T. A., Garuti R., Zhao Z., McDonald M., Horton J. D., Cohen J. C. and Hobbs H. H. (2007). Binding of proprotein convertase subtilisin/kexin type 9 to epidermal growth factor-like repeat A of low density lipoprotein receptor decreases receptor recycling and increases degradation. *J. Biol. Chem.* 282, 18602-18612. 10.1074/jbc.M702027200 17452316

